# Supplementary material for: Canonical Discriminant Mapping of Origins in Andalusian Black Cattle: Inbreeding and Coancestry Decomposition via Mendelian Sampling Variances and Nodal Ancestor Contributions
Source: Animals (Basel). 2025 Jun 17;15(12):1781. doi: 10.3390/ani15121781 (PMC12189330; doi:10.3390/ani15121781)
Supplement: Supplementary file 1 [file animals-15-01781-s001.zip › animals-3667265-supplementary.pdf]

Table S1. List of the Provinces and Municipalities where Andalusian Black Cattle individuals were born.

Province	Municipality
Córdoba	Cardeña
Córdoba	Almodóvar Del Rio
Córdoba	Cabra
Córdoba	Belmez
Córdoba	Viso (El)
Córdoba	Espiel
Córdoba	Villanueva De Córdoba
Córdoba	Carcabuey
Huelva	Cala
Huelva	Hinojos
Huelva	Alájar
Huelva	Zufre
Huelva	Cerro De Andévalo (El)
Huelva	Santa Olalla Del Cala
Jaén	Baños De La Encina
Seville	Guillena
Seville	Constantina
Seville	Cazalla De La Sierra
Seville	Castillo De Las Guardas (El)
Seville	Alcalá De Guadaira
